# SHBG and APOA1 as serum biomarkers for low bone mineral density in Mexican postmenopausal women: a random forest-based analysis

**DOI:** 10.3389/fendo.2026.1824756

**Published:** 2026-08-05

**Authors:** Diana I Aparicio-Bautista, Adriana Becerra-Cervera, Berenice Rivera-Paredez, Rogelio F Jiménez-Ortega, Tania V López-Pérez, Jorge Salmerón, J. Pablo Reyes-Grajeda, Alberto Hidalgo-Bravo, Rafael Velázquez-Cruz

**Affiliations:** 1 Laboratorio de Genómica del Metabolismo óseo, Instituto Nacional de Medicina Genómica (INMEGEN), Ciudad de México, Mexico; 2 Secretaría de Ciencias, Humanidades, Tecnología e Innovación (SECIHTI), Ciudad de México, Mexico; 3 Centro de Investigación en Políticas, Población y Salud, Facultad de Medicina, Universidad Nacional Autónoma de México, Ciudad de México, Mexico; 4 Servicio de Genómica, Instituto Nacional de Rehabilitación, Luis Guillermo Ibarra (INRLGII), Ciudad de México, Mexico; 5 Laboratorio de Estructura de Proteínas, Instituto Nacional de Medicina Genómica (INMEGEN), Ciudad de México, Mexico

**Keywords:** ApoA1, biomarkers, bone mineral density, postmenopausal women, serum, SHBG

## Abstract

**Background:**

Osteoporosis is a major health issue that has implications worldwide, both clinically and socioeconomically. Therefore, searching for informative bone health biomarkers that provide early information on bone changes and assess treatment efficacy is essential. Previously, our group used label-free proteomics and bioinformatic approaches to analyze the serum proteomic profile of postmenopausal women with normal bone mineral density (BMD), osteopenia, and osteoporosis. Based on their role in bone metabolism and literature review, a panel of 12 candidate serum biomarkers for low BMD was proposed.

**Objective:**

To validate a set of 6 proteins as potential minimally invasive serum biomarkers, Gelsolin (GSN), Sex Hormone-Binding Globulin (SHBG), Apolipoprotein A1 (APOA1), Apolipoprotein A2 (APOA2), Ryanodine Receptor 1 (RYR1), and Ryanodine Receptor 3 (RYR3), for diagnosing low BMD in postmenopausal women. We also investigated whether a combination of these proteins can provide more informative insights into the risk of having low BMD compared to individual biomarkers.

**Methods:**

The enzyme-linked immunosorbent assay was carried out to measure the concentrations of these proteins in 456 serum samples from postmenopausal Mexican women with normal or low BMD.

**Results:**

A random forest classifier demonstrated that SHBG and APOA1 are the most effective biomarkers for discriminating between women with normal or low BMD, with notable high sensitivity (86%), specificity (75%), precision (92%), and overall performance (89.32%).

**Conclusions:**

The combination of APOA1 and SHBG correlates better with the risk of bone loss than the performance of single biomarkers in postmenopausal women. Further validation in a larger cohort is necessary to confirm these results.

## 1 Introduction

Osteoporosis (OP) is characterized by a gradual loss of bone density and mass, resulting in deterioration of bone tissue, disruption of the bone microarchitecture, and weakened bone strength. These changes increase the risk of fractures, which can significantly impact quality of life, potentially resulting in physical disabilities, chronic pain, and increased mortality rates (1, 2). Although OP affects both men and women, it is particularly common in postmenopausal women due to estrogen deficiency. This deficiency leads to a significant increase in bone resorption, driven by heightened activity of osteoclasts (3).

OP is a major public health concern with clinical and socioeconomic implications worldwide. In 2010, the estimated financial cost of over 75,000 fragility fractures in Mexico was 256.2 million dollars. This number is projected to increase to around 350,000 cases by 2030, underscoring the urgent need to address this issue and prevent further adverse impacts on individuals and healthcare systems (4, 5).

Low bone mineral density (BMD) is a crucial indicator of fracture risk in patients with OP. Dual-energy X-ray absorptiometry (DXA) is a widely used clinical technique for measuring BMD (6) and is considered the gold standard for diagnosing OP. This is mainly due to its lower radiation exposure and quick scan acquisition. However, errors can occur during both the acquisition and interpretation of BMD data, which may lead to misdiagnosis (7, 8). Despite being commercially available for over three decades, DXA is often hindered by high costs and limited availability, particularly in less developed countries, restricting access for a large portion of the population (9–11).

Bone turnover biomarkers, such as C-terminal telopeptide of type 1 collagen and serum procollagen type 1 N propeptide, are recommended for clinical use to measure bone formation and resorption, respectively (12, 13). Current guidelines on OP management state that these bone turnover markers can help monitor the response to OP treatment. However, its use for diagnosing OP should still be taken with caution since studies analyzing sensitivity and specificity are still limited, and variables such as age, sex, and ethnicity can influence these markers (3, 14). Therefore, developing new and effective strategies for early OP detection and assessing fracture risk is crucial.

A biomarker is a measurable indicator of a specific biological state or clinical condition. Ideally, biomarkers should be obtained through noninvasive methods, such as those found in urine, serum, and saliva, and are characterized by their high sensitivity, specificity, precision, and reliable predictive values. In OP, biomarkers can provide advantages in monitoring the progression of the disease before a fracture occurs (15). The ongoing advancement of high-throughput detection techniques, such as proteomics, offers valuable support for screening potential disease biomarkers. Quantitative proteomics methods are beneficial for the early detection of biomarkers associated with OP and for assessing bone health. Additionally, proteomics is more effective at identifying molecular markers related to different phenotypes (16–19).

Previously, we have identified a set of serum proteins altered in postmenopausal Mexican women with low BMD using label-free LC-MS/MS quantitative proteomics and bioinformatic analyses. The proteins identified include Ryanodine receptor 1 (RYR1), Mannan-binding lectin serine protease 1 (MASP1), Sex hormone-binding globulin (SHBG), Protein-tyrosine kinase 2-beta (PTK2B), the Isoform LMW of Kininogen-1 (KNG1), Apolipoprotein A-I (APOA1), Gelsolin (GSN), Fetuin-B (FETB), Beta-2-microglobulin (B2MG), Apolipoprotein A-II (APOA2), Hemoglobin subunit delta (HBD), and Ryanodine receptor 3 (RYR3). This set of proteins was carefully selected based on an in-depth analysis of protein interactions and an extensive review of the literature. These proteins are crucial in bone metabolism and calcium pathways and are essential for maintaining optimal bone health and function (20).

Numerous studies have linked the proteins GSN, APOA1, and HBD to low BMD, while SHBG has been associated with an increased risk of fractures (16, 21–24). However, most of these studies are still in the early stages of development. Before clinical application, the last step in the biomarker discovery pipeline is to validate protein biomarkers in large human cohorts.

This study aimed to validate the novel candidate proteins GSN, RYR1, RYR3, SHBG, APOA1, and APOA2 as biomarkers for low BMD in Mexican postmenopausal women using enzyme-linked immunosorbent assay (ELISA). These novel protein biomarkers could be clinically useful for early OP diagnosis, providing valuable insights that may inform further research on bone health.

## 2 Materials and methods

### 2.1 Study population

This study is a cross-sectional analysis using a subsample from the third wave (2017–2019) of the Health Worker Cohort Study (HWCS). The HWCS is an open prospective cohort designed to examine the association between lifestyle and genetic factors and chronic diseases. The participants included employees of the Instituto Mexicano del Seguro Social (IMSS) and their relatives residing in urban areas of central Mexico. For the present analysis, we selected 456 unrelated postmenopausal women.

The characteristics of postmenopausal women have been previously reported (22).

Women were selected based on the following inclusion criteria: ≥45 years old, menopause confirmed (12 consecutive months without menstruation), and available BMD measurements. Participants were excluded if they had diabetes mellitus, chronic liver diseases, rheumatoid arthritis, collagen diseases, endocrine disorders that affect bone metabolism (such as parathyroid, gonadal, adrenal, or thyroid diseases), or if they were taking corticosteroids, anticonvulsants, bisphosphonates, or hormone replacement therapy. The measurement of the variables included in the analyses is described in detail elsewhere (25).

### 2.2 Bone mineral density measurement and osteoporosis diagnosis

Hip, femoral neck, and lumbar spine BMD (g/cm2) was measured using a Lunar DPX NT DXA instrument (Lunar Radiation Corp., Madison, WI, USA). The 456 postmenopausal women were classified by hip T-score according to the World Health Organization criteria (6, 26). The NBMD group comprised of 213 women with T-scores ranging from -1.0 and +1.0. In contrast, the LBMD group included 212 women classified as osteopenic (T-scores from -1.0 to -2.5) and 31 diagnosed with OP (T-scores below -2.5).

### 2.3 Serum sample preparation

Blood samples were collected from all participants following an 8-hour fasting period to ensure accurate metabolic measurements. The samples were then centrifuged at 2,643 x g for 15 minutes. After centrifugation, the serum was extracted, aliquoted, and stored at -80 °C until further analysis.

### 2.4 Serum biomarker measurement by ELISA assays

The concentration of GSN, SHBG, APOA1, APOA2, RYR1, and RYR3, were measured in the serum from postmenopausal women using commercial ELISA assays: GSN (Cat. NBP2-82209) biological Co., Centennial CO, USA, SHBG (Cat. EH421RB), APOA1 (Cat. EHAPOA1) and APOA2 (Cat. EH33RB) from Thermo Fisher Scientific, Waltham, MA, USA; RYR1 (Cat. MBS762816) and RYR3 (Cat. MBS166503) from My BioSource, San Diego, CA, USA. Measurements were performed according to the manufacturer’s instructions. Briefly, standards and samples were incubated in 96-well plates pre-coated with specific antibodies. Following the incubation and washing steps, a second antibody conjugated with horseradish peroxidase was added to each well. After a second incubation and washing step, tetramethylbenzidine was added as the substrate. Finally, an acidic stopping solution was added. The degree of enzymatic turnover of the substrate was determined by wavelength absorbance measurement at 450 nm using a Microplate Reader Epoch VT-05404-0998 (BioTek Instruments, Inc., Agilent, Santa Clara, CA, USA). The serum dilutions were standardized previously (GSN 1:2500, RYR1 1:10, SHBG 1:2000, APOA1 1:1,000,000, APOA2 1:100,000 and RYR3 was not diluted). Quality control procedures were implemented to ensure the accuracy and reliability of the measurements. Positive and negative controls provided by the manufacturers were included on each plate, along with standard curves to verify assay performance. Some assays were performed in duplicate to assess intra-assay variability. Inter-assay coefficients of variation (CVs) remained below 10% for all biomarkers. Only plates with control values within the manufacturer-specified range and those sample sera that passed quality control were considered for further analysis.

### 2.5 Statistical analysis

Protein concentrations were log10-transformed before analysis. The normality of continuous variables was assessed using the Shapiro–Wilk test. Based on the distribution of the data, comparisons between independent groups were performed using either the Student’s t-test (for normally distributed data) or the non-parametric Mann–Whitney U test (for non-normally distributed data). Categorical variables were compared using the Chi-square test.

Logistic regression analysis was performed to evaluate the association between protein tertiles and LBMD. Protein concentrations were categorized into tertiles, and the lowest tertile was used as the reference category. Three models were constructed: Model 1 was unadjusted; Model 2 was adjusted for age and body mass index (BMI); and Model 3 was additionally adjusted for alanine aminotransferase (ALT), aspartate aminotransferase (AST), HDL cholesterol, uric acid, serum 25(OH)D concentration, total energy intake, calcium intake, vitamin D intake, and calcium supplementation. These covariates were selected *a priori* based on their established or plausible associations with bone metabolism, nutritional status, and metabolic health. Potential multicollinearity among variables included in the fully adjusted model was evaluated using Spearman correlation coefficients and variance inflation factors (VIFs).

To further evaluate the relationship between the identified proteins and bone health, multivariable linear regression analyses were performed using hip, femoral neck, and lumbar spine bone mineral density (BMD, g/cm²) as continuous outcomes. The models were adjusted for age, BMI, ALT, AST, HDL cholesterol, uric acid, serum vitamin D concentration, total energy intake, calcium intake, vitamin D intake, and calcium supplementation.

The multiple comparisons across the protein biomarkers, the Benjamini–Hochberg procedure was implemented to control the False Discovery Rate (FDR) at a 5% significance level. This correction was applied to all between-group comparisons, tertile-based analyses, and multivariable logistic regression models involving the six proteins. FDR-adjusted *p*-values are reported in the corresponding tables, and statistical significance was defined as *p* < 0.05 to minimize the risk of Type I errors arising from simultaneous hypothesis testing.

A receiver operating characteristic (ROC) curve was estimated, and a cutoff value was obtained for the best-discriminated women with LBMD (osteopenia/osteoporosis) from NBMD postmenopausal women.

The Random Forest classifier (RF) was used to determine the ability to classify participants into their respective groups based on biomarker values. For the model prediction, the following variables were included: age, BMI, ALT, AST, HDL, uric acid, serum 25(OH)D concentration, vitamin D intake, total energy intake, calcium intake, and calcium supplementation. Two-thirds of the data were randomly selected to create the training set, while the remaining one-third was used for the validation step. The Out-of-bag (OOB) error was estimated using the training set. A confusion matrix was implemented on the validation dataset to evaluate sensitivity, specificity, positive predictive value (PPV), and negative predictive value (NPV). Logistic regression and random forest methods were implemented in R (version 4.4.1) using the random Forest and caret packages. Spearman correlation was performed between each protein and clinical information. The statistical analyses were performed using Stata version 13.0 software (Stata Corp LP, College Station, TX, USA).

## 3 Results

### 3.1 Baseline characteristics of the cohort

The baseline characteristics of the women included in the study are detailed in **Table 1**. Women in the LBMD group were significantly older, had a lower body mass index (BMI), and had lower concentrations of uric acid and ALT (*p* < 0.05). However, they had higher calcium supplement intake and HDL concentrations than women in the NBMD group (*p* < 0.01).

**Table 1:** Demographic and clinical characteristics of the study population.

Clinical and Biochemical	NBMD, n=213	LBMD, n=243	*p*-value
Characteristics	Median (P25-P75)	Median (P25-P75)	
Age, years	49 (37-58)	66 (59-73)	5.1x10^-33^
RYR1, ng/mL	8.7 (7.5-10.2)	8.5 (7.1-10.8)	0.8122
GSN, ug/mL	177 (151-220)	196 (152-273)	0.0067
SHBG, nmol/L	33.5 (23.3-51.9)	52.5 (39.5-70.4)	5x10^-15^
APOA1, ug/mL	278 (150-658)	472 (240-1302)	0.0004
APOA2, mg/dL	27.3 (21.7-36.4)	30.2 (23.5-38.1)	0.0834
RYR3, ng/mL	2.9 (1.9-5.0)	2.7 (2.1-3.8)	0.379
Hip BMD, g/cm^2^	1.087 (1.044-1.152)	0.800 (0.748-0.833)	<0.001
Hip T-score	0.632 (0.2919-1.146)	-1.646 (-2.062,-1.379)	<0.001
BMI, kg/cm^2^	29.1 (25.8-33.0)	25 (22.7-27.8)	3.5x10^-16^
Overweigth, %	37.6	37.5	0.9807
Obesity, %	43.2	13.2	1.5x10^-12^
Calcium intake, mg/day	833 (566-1239)	804 (566-1178)	0.5386
Vitamin D intake, UI/day	111 (65-170)	103 (69-158)	0.3891
Calcium supplements, %	7.8	26.8	1.3x10^-7^
25 (OH)D, ng/mL	24.6 (19.5-30.4)	24.6 (19.8-29.4)	0.6263
Vitamin D deficiency, %	26.8	25.9	0.8276
HDL, mg/dL	51 (41-60)	54 (47-67)	0.0007
Uric acid, mg/dL	5 (4.4-6.1)	4.8 (4.1-5.7)	0.0238
ALT, U/L	26 (20-36)	22 (17-29)	0.0001
AST, U/L	24 (20-31)	25 (22-30)	0.3343

NBMD, Normal-Bone Mineral Density; LBMD, Low-Bone Mineral Density; RYR1, Ryanodine receptor 1; RYR3, Ryanodine receptor 3; SHBG, Sex Hormone Binding Globulin; APOA1, Apolipoprotein A-I; APOA2, Apolipoprotein A-II; BMD, Bone Mineral Density; BMI, Body Mass Index; 25 (OH)D, 25-hydroxyvitamin D; HDL, High-Density Lipoprotein; ALT, Alanine aminotransferase; AST, Aspartate aminotransferase. The U Mann-Whitney test was used for continuous variables, and the Chi-square test was used for categorical variables. *p* < 0.05 was considered statistically significant. Data are expressed as the median (inter-quartile range) or percentage.

### 3.2 Validation of serum biomarkers associated with low BMD

**Figure 1:**
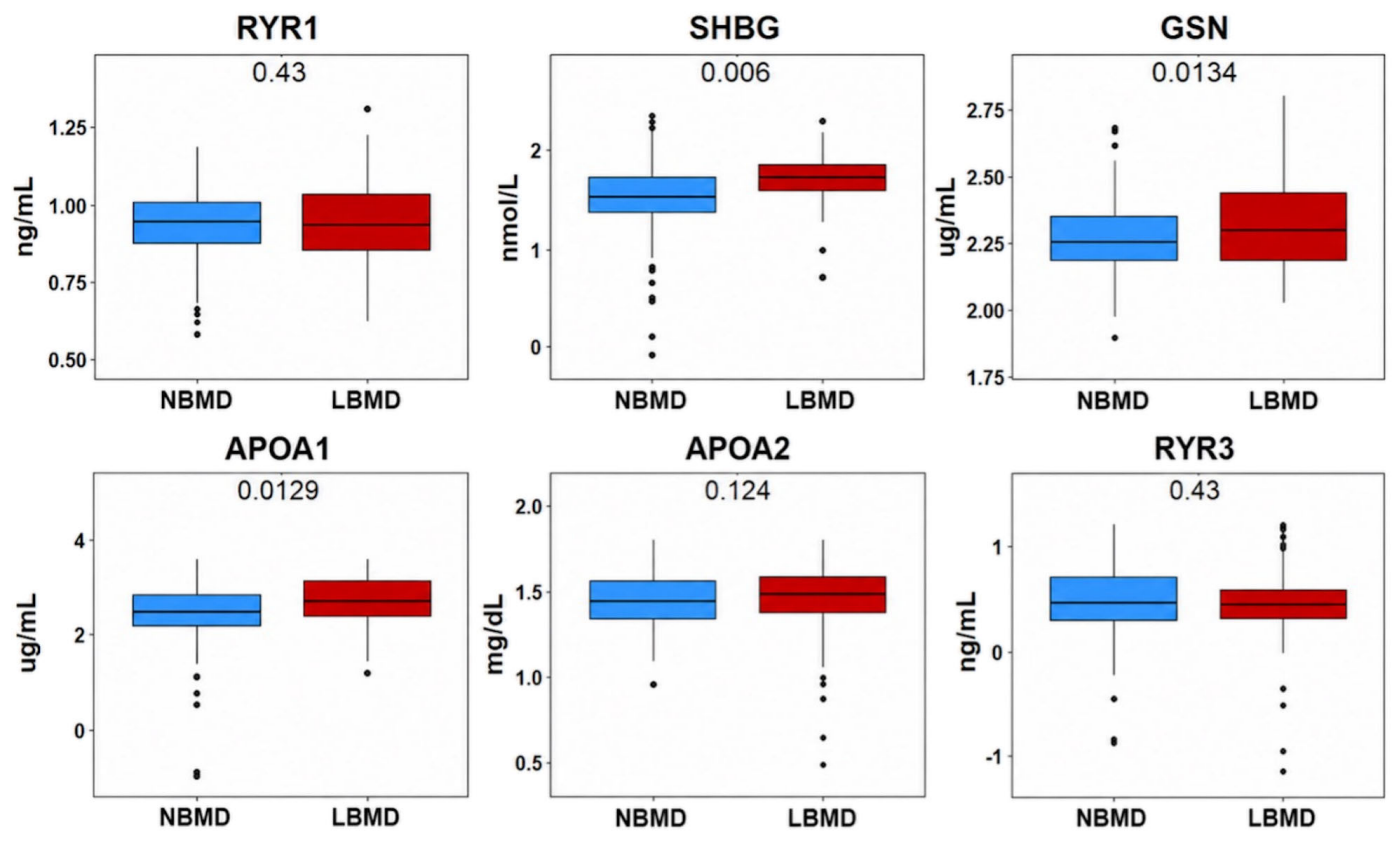
The comparison of serum concentrations of RYR1, SHBG, APOA1, APOA2, GSN, and RYR3 proteins between the NBMD and LBMD groups. Data were log10-transformed and analyzed using the Mann–Whitney U test. NBMD, Normal-Bone Mineral Density; LBMD, Low-Bone Mineral Density.

Statistical analysis revealed significantly higher concentrations of APOA1 (*p* = 0.0129), GSN (*p* = 0.0134), and SHBG (*p* = 0.006) in the LBMD group. In contrast, no significant differences were observed in the concentrations of APOA2, RYR1, and RYR3 between groups (**Figure 1**). ROC analysis with raw data showed that SHBG had the highest discriminatory ability, with an AUC of 0.741 (95% CI: 0.684-0.799) and a cutoff value of 35.68 ng/mL for the detection of LBMD. APOA1 and GSN demonstrated limited performance (AUC = 0.553 and 0.551), with cutoff values of 178.09 mg/dL and 216.77 ng/mL, respectively. RYR1, RYR3, and APOA2 showed no discriminatory value (AUC <0.5). These results suggest that SHBG has potential as a diagnostic biomarker (**Supplementary Figure 1**).

### 3.3 Association between serum biomarker concentrations and BMD status

**Figure 2:**
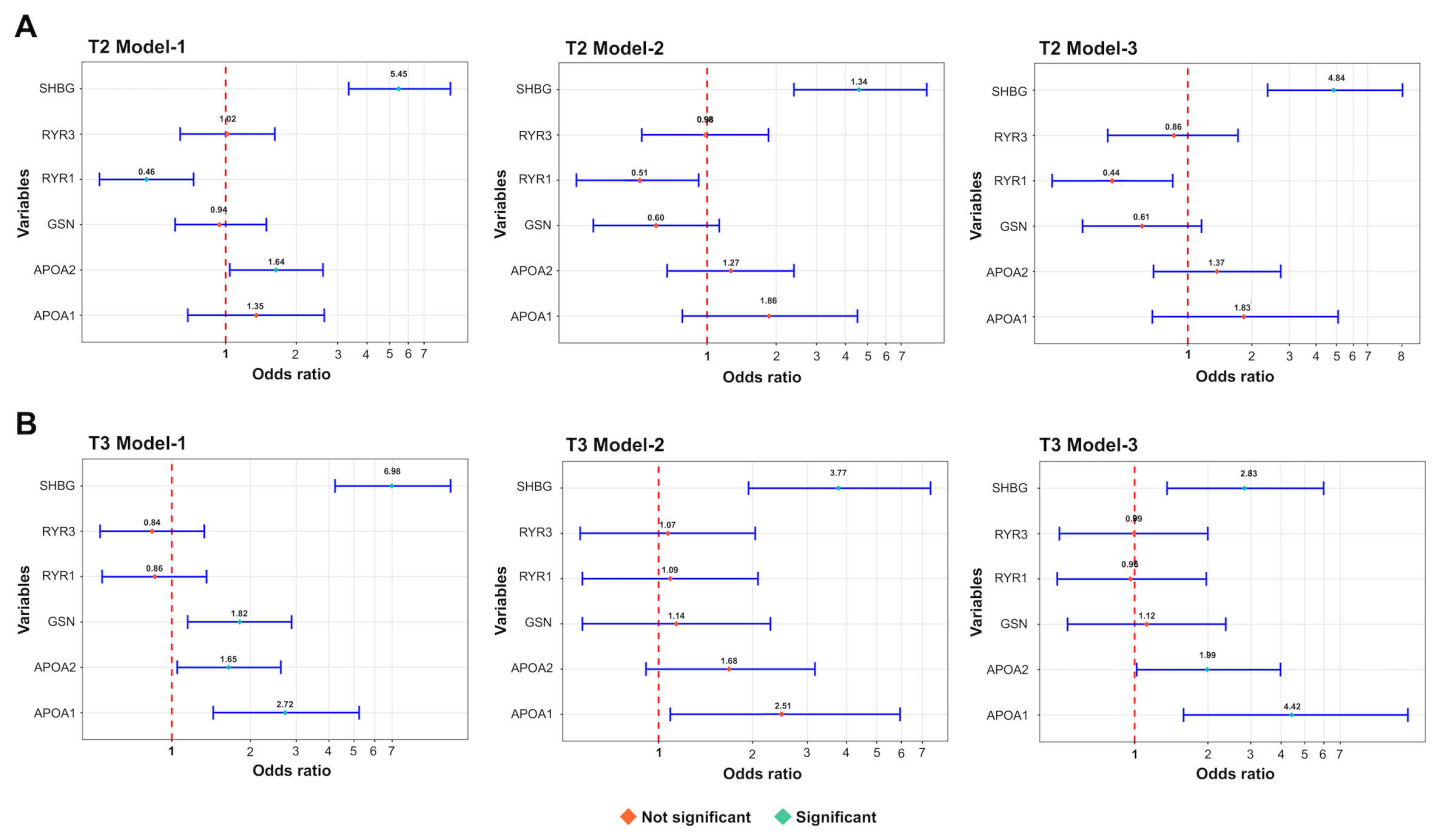
Forest plot of logistic regression analysis for protein tertiles (Tertile 2 [T2] and Tertile 3 [T3]) in relation to low BMD. The reference category was Tertile 1 of each protein. Panel **(A)** 1) Comparison between Tertile 2 and Tertile 1 for proteins associated with low BMD, unadjusted. 2) Comparison between Tertile 2 and Tertile 1 for proteins associated with low BMD, adjusted for age and BMI. 3) Comparison between Tertile 2 and Tertile 1 for proteins associated with low BMD, adjusted for age, BMI, ALT, AST, HDL, uric acid, serum vitamin D concentration, total energy intake, calcium intake, vitamin D intake and calcium supplementation. Panel **(B)**: 4) Comparison between Tertile 3 and Tertile 1 for proteins associated with low BMD, unadjusted. 5) Comparison between Tertile 3 and Tertile 1 for proteins associated with low BMD, adjusted for age and BMI. 6) Comparison between Tertile 3 and Tertile 1 for proteins associated with low BMD, adjusted for age, BMI, ALT, AST, HDL, uric acid, serum vitamin D concentration, total energy intake, calcium intake, vitamin D intake, and calcium supplementation. The red line represents the reference. Odds ratios (OR) above 1 are associated with higher odds of low BMD, while ORs below 1 are protective against low BMD. 95% confidence intervals (CI) that do not cross the red line are statistically significant, *p* < 0.05. BMI, Body Mass Index; 25(OH)D, 25-hydroxyvitamin D; HDL, High-Density Lipoprotein; ALT, Alanine aminotransferase; AST, Aspartate aminotransferase. FDR-adjusted *p*-values.

Logistic regression analysis examined the relationship between protein concentration tertiles and BMD status, adjusted for three models (**Table 2**; **Figure 2**). RYR1 showed a protective effect against LBMD in T2 compared to T1 under model 1 (OR 0.46, 95% CI 0.29-0.73, *p* < 0.01). In contrast, the comparison between T3 and T1 under models 1, 2, and 3 was not significant. A significant association was observed between SHBG and an increased odds of low BMD in T2 and T3 compared to the lowest tertile across all models (*p* < 0.05). APOA1 was significantly associated with a higher risk of low BMD (*p* < 0.05), with individuals in T3 having 2.72 and 4.50 times the odds of low BMD compared to those in T1, as shown in models 1 and 3, respectively. GSN showed greater odds for low BMD in T3 versus T1 under model 1 (*p* < 0.05). APOA2 showed borderline significance in models T2 and T3 compared with T1 (*p* = 0.049). RYR3 did not show any statistically significant association. These results underscore the potential of SHBG and APOA1 as biomarkers for predicting LBMD.

**Table 2:** Association between protein concentrations and BMD status.

Protein	Tercil	NBMD	LBMD	Model 1		Model 2		Model 3	
		n	n	OR (95%CI)	Adjusted *pvalue*	OR (95%CI)	Adjusted *pvalue*	OR (95%CI)	Adjusted *pvalue*
RYR1	T1	60	93	1.0		1.0		1.0	
	T2	88	63	0.46 (0.29, 0.73)	1.80E-3	0.51 (0.27, 0.92)	0.056	0.444 (0.23, 0.85)	0.08
	T3	65	87	0.86 (0.54, 1.36)	0.529	1.09 (0.57, 2.08)	0.800	0.95 (0.47, 1.93)	1.00
SHBG	T1	111	38	1.0		1.0		1.0	
	T2	52	97	5.45 (3.34, 9.07)	4.20E-11	4.59 (2.39, 9.05)	1.31E-05	4.84 (2.37, 10.22)	1.06E-4
	T3	44	195	6.98 (4.23, 11.73)	2.41E-13	3.77 (1.94, 7.44)	1.04E-03	2.68 (1.28, 5.62)	0.014
GSN	T1	77	74	1.0		1.0		1.0	
	T2	79	72	0.94 (0.61, 1.49)	1.000	0.6 (0.32, 1.13)	0.218	0.61 (0.32, 1.16)	0.665
	T3	55	96	1.82 (1.15, 2.88)	0.033	1.14 (0.57, 2.28)	0.718	1.13 (0.53, 2.40)	1.0
APOA1	T1	61	23	1.0		1.0		1.0	
	T2	55	28	1.35 (0.69, 2.63)	0.560	1.86 (0.78, 4.52)	0.1646	1.83(0.68, 5.08)	0.585
	T3	41	42	2.72 (1.44, 5.23)	0.007	2.51 (1.09, 5.94)	0.0640	4.50 (1.55,13.00)	0.030
APOA2	T1	82	67	1.0		1.0		1.0	
	T2	64	86	1.64 (1.04, 2.6)	0.049	1.27 (0.67, 2.39)	0.569	1.37(0.69, 2.73)	0.925
	T3	63	85	1.65 (1.05, 2.62)	0.049	1.68 (0.91, 3.17)	0.208	2.24 (1.11, 4.51)	0.235
RYR3	T1	68	82	1.0		1.0		1.0	
	T2	64	79	1.02 (0.64, 1.62)	1.00	0.98 (0.52, 1.85)	1.00	0.8(0.42, 1.72)	1.0
	T3	73	74	0.84 (0.53, 1.33)	1.00	1.07 (0.56, 2.04)	1.00	0.99 (0.49, 2.00)	0.08

### 3.4 Association between serum biomarker tertiles and BMD at different skeletal sites

Associations between protein tertiles and site-specific BMD were further evaluated using multivariable linear regression models (**Supplementary Table 1**). Higher SHBG concentrations were significantly associated with lower BMD at the hip and femoral neck. Compared with women in the lowest tertile, those in the second tertile had lower hip BMD (β = −0.059, 95% CI: −0.090 to −0.028, *p* = 0.0012) and femoral neck BMD (β = −0.049, 95% CI: −0.079 to −0.020, *p* = 0.012). Similar associations were observed for the third tertile (hip BMD: β = −0.056, 95% CI: −0.089 to −0.022, *p* = 0.006; femoral neck BMD: β = −0.043, 95% CI: −0.075 to −0.011, *p* = 0.036). GSN was marginally associated with hip and femoral neck BMD in the second tertile (hip BMD: β = 0.038, 95% CI: 0.008 to 0.069, *p* = 0.056; femoral neck BMD: β = 0.040, 95% CI: 0.011 to 0.070, *p* = 0.042). No significant associations were observed between APOA1, APOA2, RYR1, or RYR3 tertiles and hip, femoral neck, or lumbar spine BMD after multivariable adjustment. Similarly, none of the evaluated proteins showed significant associations with lumbar spine BMD.

### 3.5 ROC curves analysis to determine biomarker clinical utility

**Figure 3:**
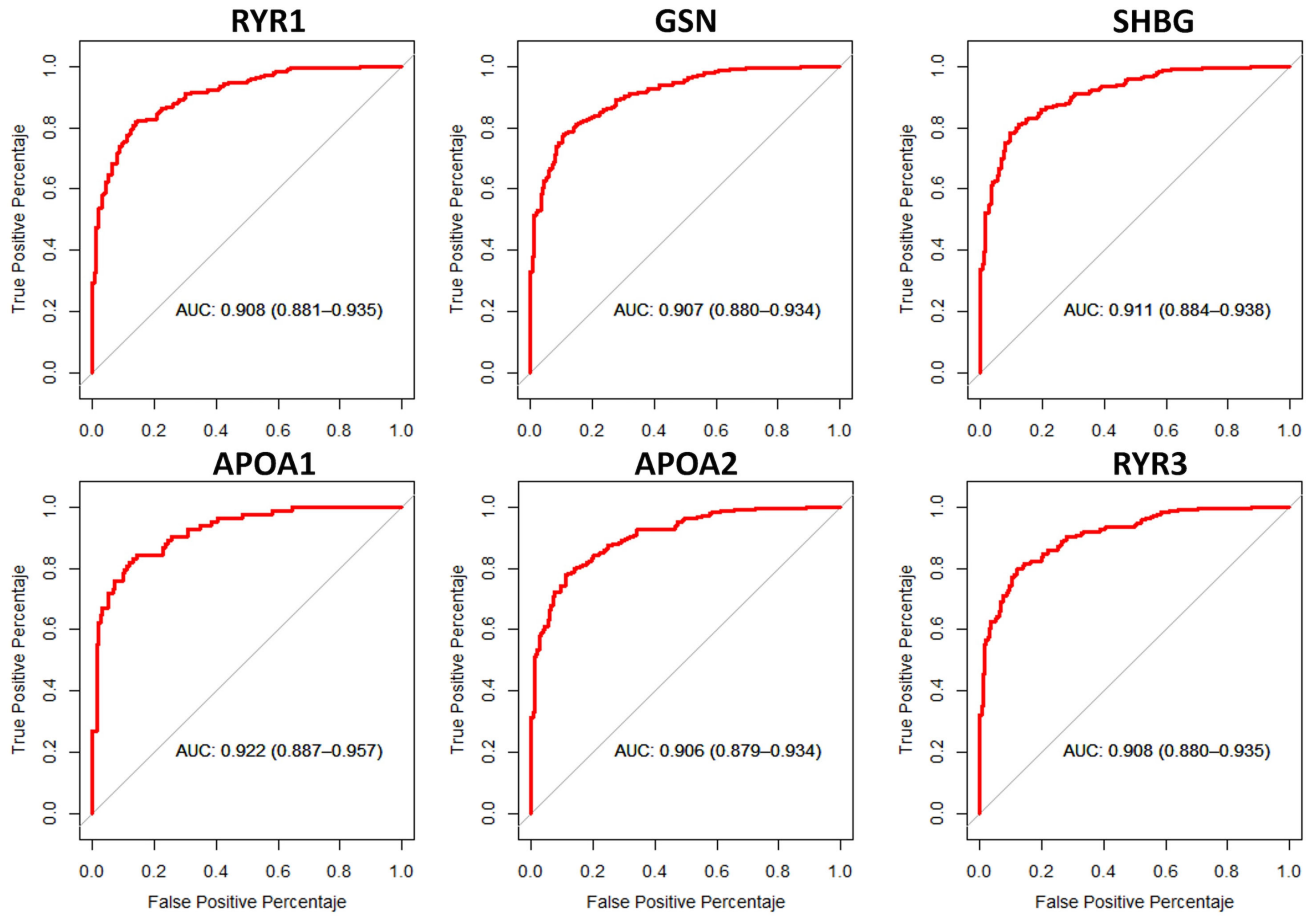
Receivers operate curves. The selected candidate biomarkers for LBMD vs. NBMD had individual AUCs > 90%. All ROC curves were generated using the protein abundance adjusted under model 3: age and BMI, ALT, AST, HDL, uric acid, serum vitamin D concentration + total energy intake, calcium intake, vitamin D intake, and calcium supplementation. NBMD, Normal-Bone Mineral Density; LBMD, Low-Bone Mineral Density; BMI, Body Mass Index; 25(OH)D, 25-hydroxyvitamin D; HDL, High-Density Lipoprotein; ALT, Alanine aminotransferase; AST, Aspartate aminotransferase. FDR-adjusted *p*-values.

A comparative analysis of three different predictive models for RYR1, GSN, SHBG, APOA1, APOA2, and RYR3 was conducted (**Table 3**). RYR1 and RYR3 showed limited predictive performance in model 1 (50.6% and 52%, respectively). Models 2 and 3 exhibited superior discriminative performance across all proteins analyzed, with AUC values above 82% (*p* < 0.001) (**Figure 3**; **Table 3**). These findings suggest that models incorporating multiple risk factors enhance biomarker-based diagnostic accuracy.

**Table 3:** ROC curves to examine the potential clinical utility of the candidate biomarkers in diagnosing low BMD.

Protein	Model	AUC (95% CI)	Adjusted *pvalue*
RYR1	Model 1	0.506(0.45-0.56)	1.0
	Model 2	0.898(0.87-0.92)	<0.001
	Model 3	0.91(0.88-0.93)	<0.001
GSN	Model 1	0.571 (0.52-0.62)	0.071
	Model 2	0.896 (0.86-0.92)	<0.001
	Model 3	0.907(0.88-093)	<0.001
SHBG	Model 1	0.71(0.66-0.76)	2.8E-3
	Model 2	0.89(0.87-0.93)	<0.001
	Model 3	0.91(0.88-0.04)	<0.001
APOA1	Model 1	0.63(0.56-0.7)	1.0
	Model 2	0.88(0.84-0.93)	<0.001
	Model 3	0.92(0.88-0.95)	<0.001
APOA2	Model 1	0.59(0.49-0.6)	0.123
	Model 2	0.89(0.87-0.92)	<0.001
	Model 3	0.91(0.88-0.93)	<0.001
RYR3	Model 1	0.52(0.47-0.58)	1.0
	Model 2	0.82(0.87-0.92)	<0.001
	Model 3	0.83(0.88-0.93)	0.0001

Model 1: without adjustment. Model 2: adjusted by age and BMI. Model 3: adjusted by age, BMI, AST, ALT, HDL, uric acid, serum vitamin D concentration, total energy intake, calcium intake, vitamin D intake and calcium supplementation. The Area Under the Curve (AUC) is shown along with its 95% confidence interval (CI). *P* value adjusted using the Benjamini–Hochberg false discovery rate (FDR) procedure.

### 3.6 Classification of NBMD and LBMD using random forest analysis

RF analysis evaluated the effectiveness of selected proteins in classifying LBMD and NBMD groups (**Table 4**). All potential biomarkers were statistically significant (*p* < 0.05). The OOB error varied among proteins, with APOA2 exhibiting the highest OOB error (20.96%) and RYR3 the lowest (18.53%). APOA1 (81.69%) and RYR1 (80.47%) achieved accuracy greater than 80%, followed by GSN (78.74%), APOA2 (78.57%), and SHBG (78.40%). RYR1 demonstrated the highest significance (*p* = 1.03 x 10^-10^), the highest specificity (83.58%), the highest accuracy (80.47%), and the highest level of agreement between predicted and observed classifications (60.75%). Regarding sensitivity, PPV, and detection rate, APOA1 outperformed others (82.35%, 91.3%, and 59.15%, respectively).

**Table 4:** Comparison of random forest model performance using selected proteins.

Statistical Metrics	RYR1	GSN	SHGB	APOA1	APOA2	RYR3
**LBMD**	220	219	217	82	216	213
**NBMD**	204	202	198	153	201	196
OOB estimate of error rate (%) *	19.59	19.73	19.66	19.51	20.96	18.53
Accuracy	**0.8047**	0.7874	0.784	**0.8169**	0.7857	0.748
95% CI	(0.7253, 0.8694)	(0.706, 0.855)	(0.7015, 0.8526)	(0.7073, 0.8987)	(0.7038, 0.8538)	(0.6617, 0.8219)
ROC-AUC (%)						
No Information Rate	0.5312	0.5512	0.536	**0.6479**	0.5476	0.5285
P-Value [Acc > NIR]	**1.03 x 10^-10^**	2.38 x 10^-8^	7.55 x 10^-9^	0.001408	2.21 x 10^-8^	4.65 x 10^-7^
Kappa	**0.6075**	0.571	0.5673	0.5795	0.5682	0.4938
Mcnemar’s Test P-Value	1	1	0.7003	0.267257	1	1
Sensitivity	0.7869	0.7719	0.7541	**0.8235**	0.7586	0.7368
Specificity	**0.8358**	0.8	0.8125	0.8	0.8088	0.7576
Pos Pred Value	0.8136	0.7586	0.7931	**0.913**	0.7719	0.7241
Neg Pred Value	**0.8116**	**0.8116**	0.7761	0.64	0.7971	0.7692
Precision	0.8136	0.7586	0.7931	**0.913**	0.7719	0.7241
Recall	0.7869	0.7719	0.7541	0.8235	0.7586	0.7368
F1-score	0.8	0.7652	0.7731	**0.866**	0.7652	0.7304
Prevalence	0.4766	0.4488	0.488	0.7183	0.4603	0.4634
Detection Rate	0.375	0.3465	0.368	0.5915	0.3492	0.3415
Detection Prevalence	0.4609	0.4567	0.464	0.6479	0.4524	0.4715
Balanced Accuracy	0.8114	0.786	0.7833	0.8118	0.7837	0.7472

Bold values indicate the greatest value for each metric. CI, confidence interval. Balanced accuracy = (sensitivity + specificity)/2. Protein concentrations were adjusted by age, BMI, ALT, AST, HDL, uric acid, serum vitamin D concentration, total energy intake, calcium intake, vitamin D intake, and calcium supplementation. *Training data was obtained using matrix confusion. NBMD, Normal-Bone Mineral Density; LBMD, Low-Bone Mineral Density.

Combinations of selected biomarkers were evaluated to enhance classification accuracy and minimize error rates. The combination of APOA1 + SHBG had the lowest error rate (18.35%) and the highest accuracy, sensitivity, and precision (84.06%, 86.70%, and 92%, respectively), with a classification performance of 89.32%. The combined RYR1 + APOA1 had the highest error rate (21.34%) but still showed high accuracy (83.1%), sensitivity (82.69%), specificity (84.21%), and precision (93.48%), resulting in an overall performance of 87.76% (**Supplementary Table 2**). These results suggest that APOA1 and SHBG can effectively classify individuals with low BMD. Additionally, the APOA1 + SHBG combination demonstrated high performance in classifying osteopenia. Among the 215 samples included in the analysis (68 cases of osteopenia and 147 controls), the model achieved an overall accuracy of 90.8% (95% CI: 80.9–96.5%). Sensitivity and specificity were 90.2% and 92.9%, respectively, with an accuracy of 97.9%, showing the ability to correctly identify both osteopenia cases and controls (**Supplementary Table 3**).

### 3.7 Correlation between clinical parameters and the potential biomarker proteins.

Hip BMD showed negative correlations with SHBG, APOA1, and GSN (*p* < 0.05). Lumbar BMD was negatively correlated with SHBG (−0.2463) and GSN (−0.0911). Likewise, femoral neck BMD showed negative correlations with SHBG (−0.3554) and APOA1 (−0.1471). The correlations between the T-scores of the hip, lumbar spine, and femoral neck with the serum biomarkers were similar to those observed with BMD variables. Specifically, GSN, SHBG, and APOA1 exhibited negative correlations. Furthermore, SHBG protein showed a positive association with HDL, whereas its associations with AST and uric acid were negative (**Table 5**). These correlations suggest an inverse relationship between SHBG and APOA1 concentrations and BMD and T-scores.

**Table 5:** Correlation matrix of clinical data versus candidate biomarker proteins.

Variable	RYR1	GSN	SHBG	APOA1	APO2	RYR3
Hip BMD	0.0067	**-0.117**	**-0.3756**	**-0.1515**	-0.0322	0.0464
Lumbar BMD	-0.0556	**-0.0911**	**-0.2463**	-0.1013	-0.0262	-0.0042
Femoral neck BMD	0.0099	-0.159	**-0.3554**	**-0.1471**	-0.029	0.0612
Hip T-score	0.0067	**-0.117**	**-0.3756**	**-0.1515**	-0.0322	0.0464
Lumbar T-score	-0.0086	**-0.1145**	**-0.2545**	**-0.1341**	-0.0247	-0.0038
Femoral neck T-score	0.0156	**-0.1516**	**-0.3641**	**-0.1446**	-0.0379	0.0575
Uric acid	-0.0098	**0.1961**	**-0.2023**	0.0101	**-0.0986**	-0.0467
AST	0.0053	**0.1893**	0.077	0.0235	-0.0419	-0.0854
ALT	0.0233	**0.0942**	**-0.1562**	0.0155	0.0021	-0.0811
HDL	-0.0633	**-0.0941**	**0.2484**	0.0693	0.0526	-0.0083

Bold values indicate *p*-value <0.05. BMD, Bone Mineral Density; HDL, High-Density Lipoprotein; ALT, Alanine aminotransferase; AST, Aspartate aminotransferase.

## 4 Discussion

The current investigation validated six proteins previously proposed as potential biomarkers associated with low BMD in postmenopausal women, specifically, RYR1, RYR3, SHBG, GSN, APOA1, and APOA2. Our findings indicate that SHBG, GSN, and APOA1 concentrations were significantly elevated in women with low BMD. The linear regression analysis revealed that SHBG, APOA1, GSN, and APOA2 were associated with an increased risk for low BMD, whereas RYR1 exhibited a protective effect. Previous studies have reported that serum concentrations of APOA1 and SHBG improve diagnostic performance for osteopenia and OP (27, 28).

The negative correlation between serum SHBG concentrations and BMD at the hip, lumbar spine, and femoral neck observed in our study is consistent with previous findings reported in both men and women (29–33). SHBG plays a crucial role in bone metabolism by binding, transporting, and regulating circulating androgens and estrogens (34). One proposed mechanism underlying SHBG’s impact on BMD is its anti-estrogenic effect; elevated SHBG concentrations reduce the bioavailability of active estrogens, leading to diminished estrogen-mediated bone protection and an increased risk of fractures (35). Recent evidence indicates that OP development is influenced not only by estrogen deficiency but also by lipid metabolism disorders (36). In this context, SHBG has been positively correlated with HDL concentrations in postmenopausal women (37). Interestingly, our study observed higher concentrations of SHBG, APOA1, and HDL associated with low BMD.

The specific role of APOA1 and its relationship with BMD remains controversial. Some studies report that APOA1 may have a protective effect on BMD, correlated with enhanced lumbar BMD and C-terminal telopeptide of type I in Chinese patients with osteoporotic fractures, suggesting that APOA1 may inhibit osteoclast activity or regulate osteoblast activity (38). In contrast, in agreement with our results, a cross-sectional study from the Third National Health and Nutrition Examination Survey found that higher concentrations of APOA1 and HDL-C increase the risk of OP in older women with high BMI, hypertension, and hypercholesterolemia, suggesting that lipid metabolism may play a role in the occurrence and development of OP (27). Even APOA1 and HDL-C concentrations are considered potential indicators for predicting osteopenia or osteoporosis (39, 40). Although APOA1 is the primary protein component and major carrier of HDL-C, our correlation analysis did not show a significant association between APOA1 and HDL-C levels. This unexpected finding may be due to the complex regulation of HDL metabolism, which involves various factors beyond APOA1 concentration, such as enzymatic activity (e.g., LCAT, CETP), hepatic clearance, and inflammatory or metabolic states (41). Additionally, differences in genetic background, lifestyle, or comorbidities in the study population may have influenced these results. These observations underscore the need for further studies to clarify the functional relationship between APOA1, HDL-C, and bone health.

The analysis of ROC curves for GSN, SHBG, APOA1, APOA2, RYR1, and RYR3 consistently resulted in AUC values exceeding 88%. However, the classifier’s metrics (accuracy, sensitivity, and precision) showed variances between these biomarkers. For example, RYR3 has the lowest error rate but lower performance in terms of sensitivity and specificity, while APOA1 has better metrics. These differences between models should be considered in the future, as the APOA1 model revealed that most participants had normal BMD (65% of NBMD vs. 35% of LBMD, n = 235). While the Random Forest analysis is robust to class imbalances, the higher proportion of participants with normal BMD may have influenced the model’s ability to correctly identify the low BMD (LBMD) class, which could affect some of the model’s metrics.

Combining several biomarkers improves the overall diagnostic performance, a common practice in clinical studies and biomedical research. In our study, the combination of APOA1 and SHBG proteins demonstrated high accuracy, sensitivity, and specificity. These findings support the potential of combining biomarkers to create diagnostic models for improving the classification of LBMD in postmenopausal women. This model could have significant implications for early diagnosis and risk stratification in clinical practice, particularly among high-risk populations with osteoporosis. It is essential to consider that the serum levels of the proteins analyzed in this study may also be influenced by other physiological or pathological conditions, which could act as confounding factors. For example, SHBG levels are known to be affected by liver function, thyroid status, and insulin resistance (42). Although we adjusted for several clinical and biochemical variables, the possibility of residual confounding due to unmeasured or undiagnosed conditions cannot be excluded. Therefore, future studies should account for these potential sources of bias to better isolate the specific relationship between these proteins and BMD.

Finally, our study revealed that age and BMI are highly relevant features for predictive models. It is well established that advanced age and female sex are the primary risk factors for osteoporosis (43). According to the WHO, most women experience natural menopause between the ages of 45 and 55. Following menopause, estrogen deficiency accelerates bone resorption, resulting in progressive bone loss and an increased risk of osteopenia and osteoporosis. Davis et al. (44) reported that menopause typically occurs at 47 years among Latin women and emphasized that both aging and prolonged exposure to estrogen deficiency are key determinants of skeletal deterioration in postmenopausal women (44). The pathophysiology of age-related osteoporosis involves changes in osteocyte density and connectivity, as well as the accumulation of senescent bone cells, which disrupt bone microstructural integrity and increase bone fragility. With advancing age, alterations in the activity of lipid metabolic enzymes disrupt osteogenesis and adipogenesis, thereby promoting bone loss (45, 46). Therefore, the older age observed in the LBMD group is biologically plausible and aligns with the natural progression of postmenopausal bone loss. However, the marked age difference between the NBMD and LBMD groups constitutes a limitation of the present study, as age is a major determinant of bone mineral density and may have influenced some of the observed associations.

Although our descriptive analysis showed that women with LBMD had significantly lower BMI and lower prevalence of obesity, the multivariate logistic regression analysis confirmed a strong inverse association between BMI category and the odds of low BMD. Specifically, compared to women with normal BMI, those in the overweight and obese categories had 90% and 98% lower odds of having LBMD, respectively. These results are consistent with previous studies suggesting that higher body weight exerts a protective effect on bone density, possibly due to increased mechanical loading and higher estrogen levels from adipose tissue (47–49). These findings suggest a complex interplay between lipid metabolism, hormonal balance, and bone health, warranting further investigation into the potential mechanisms linking SHBG, RYR1 and APOA1 to osteoporosis risk.

This study has several strengths: (i) This is the first study on postmenopausal Mexican women, including a large cohort with complete clinical information. (ii) The ROC curves and FR analyses demonstrated that all selected proteins have a statistically significant impact on predicting BMD. This study has several limitations: (i) the sample was drawn exclusively from healthcare workers in central Mexico. As a result, participants may have greater baseline knowledge about bone health than the general population, potentially introducing selection bias. Additionally, genetic, cultural, and environmental differences may limit the applicability of the observed associations between these proteins and BMD to other regions or ethnic groups. (ii) Individuals with common medical conditions, such as diabetes, liver disease, and rheumatoid arthritis, were excluded. Therefore, the study population may not be representative of the general population of postmenopausal women, particularly those with metabolic comorbidities or systemic diseases. (iii) Although the combined models show potential, their practical application requires validation in larger, more diverse cohorts to ensure generalizability across broader clinical populations (iv) Although the study possessed sufficient statistical power to assess differences between individuals with NMBD and LBMD, the small number of osteoporosis cases limited the ability to conduct stratified analyses by disease severity. Therefore, it was not possible to determine whether the diagnostic performance of the SHBG+APOA1 biomarker panel differs between osteopenia and osteoporosis or between NMBD and osteoporosis. Further research with larger prospective cohorts is necessary to address this limitation. (v) Although the age distribution in this cohort aligns with the epidemiology of postmenopausal osteoporosis, the considerable age difference between groups represents a limitation of the study. Because aging significantly influences bone metabolism, some observed variability may result from age-related biological changes. Future studies involving age-matched cohorts are necessary to further validate these findings. (vi) The discrepancy observed in RYR1 between the discovery and validation stages could be resolved by employing highly sensitive and specific techniques, such as multiple reaction monitoring. (vii) Further research is needed to identify how these biomarkers influence bone health and investigate how lipid metabolism, calcium signaling, and hormone regulation interact to impact BMD in postmenopausal women.

## 5 Conclusions

The present study highlights the potential of specific biomarkers, particularly SHBG and APOA1, for diagnosing low BMD in postmenopausal Mexican women. Complex models combining multiple biomarkers demonstrated superior performance, improving both diagnostic accuracy and sensitivity. These results underscore the need for integrating metabolic, hormonal, and genetic factors into predictive models for osteoporosis, which could ultimately enhance early detection and personalized treatment strategies. Further research will be necessary to refine these models and explore their clinical utility in diverse populations.

T1: Tertile 1, T2: Tertile 2, T3: Tertile 3. Model 1: without adjustment. Model 2: adjusted by age and BMI. Model 3: age, BMI, ALT, AST, HDL, uric acid, serum vitamin D concentration, total energy intake, calcium intake, vitamin D intake and calcium supplementation. NBMD, Normal-Bone Mineral Density; LBMD, Low-Bone Mineral Density; BMI, Body Mass Index; HDL, High-Density Lipoprotein; ALT, Alanine aminotransferase; AST, Aspartate aminotransferase. *P* values were adjusted using the Benjamini–Hochberg false discovery rate (FDR) procedure.

## Data Availability

The original contributions presented in the study are included in the article/**Supplementary Material**. Further inquiries can be directed to the corresponding author.
