# Supplementary material for: SHBG and APOA1 as serum biomarkers for low bone mineral density in Mexican postmenopausal women: a random forest-based analysis
Source: Front Endocrinol (Lausanne). 2026 Aug 5;17:1824756. doi: 10.3389/fendo.2026.1824756 (PMC13485816; doi:10.3389/fendo.2026.1824756)
Supplement: Supplementary file 1 [file Table1.docx]

**Supplemental material**

**Supplementary Table 1.** Multivariable linear regression analyses of the associations between protein tertiles and bone mineral density at the hip, femoral neck, and lumbar spine in postmenopausal women.

| **Protein** | **Tercil** | **Hip BMD** | | **Femoral neck BMD** | | **Lumbar spine BMD** | |
| --- | --- | --- | --- | --- | --- | --- | --- |
|  |  | **β (95%CI)** | **Adjusted**  ***pvalue*** | **β (95%CI)** | **Adjusted**  ***pvalue*** | **β (95%CI)** | **Adjusted**  ***pvalue*** |
| **RYR1** | T1 | 0 |  | 0 |  | 0 |  |
|  | T2 | 0.031 (0.001,0.061) | 0.132 | 0.021 (-0.008,0.049) | 0.483 | 0.009 (-0.027,0.046) | 0.819 |
|  | T3 | -0.001 (-0.031,0.030) | 0.967 | -0.009 (-0.038,0.021) | 0.612 | -0.023 (-0.060,0.015) | 0.47 |
| **SHBG** | T1 | 0 |  | 0 |  | 0 |  |
|  | T2 | -0.059 (-0.090,-0.028) | 0.0012 | -0.049 (-0.079,-0.020) | 0.012 | -0.029 (-0.067,0.009) | 0.384 |
|  | T3 | -0.056 (-0.089,-0.022) | 0.006 | -0.043 (-0.075,-0.011) | 0.036 | -0.020 (-0.061,0.021) | 0.591 |
| **GSN** | T1 | 0 |  | 0 |  | 0 |  |
|  | T2 | 0.038 (0.008,0.069) | 0.056 | 0.040 (0.011,0.070) | 0.042 | 0.035 (-0.002,0.073) | 0.384 |
|  | T3 | 0.015 (-0.018,0.047) | 0.578 | 0.018 (-0.014,0.049) | 0.453 | 0.039 (-0.001,0.079) | 0.684 |
| **APOA1** | T1 | 0 |  | 0 |  | 0 |  |
|  | T2 | -0.019 (-0.059,0.022) | 0.626 | -0.011 (-0.051,0.028) | 0.567 | 0.009 (-0.042,0.059) | 0.884 |
|  | T3 | -0.025 (-0.067,0.016) | 0.557 | -0.023 (-0.064,0.017) | 0.51 | -0.023 (-0.075,0.029) | 0.573 |
| **APOA2** | T1 | 0 |  | 0 |  | 0 |  |
|  | T2 | -0.013 (-0.043,0.018) | 0.564 | -0.010 (-0.040,0.020) | 0.614 | 0.004 (-0.033,0.041) | 0.920 |
|  | T3 | -0.018 (-0.050,0.014) | 0.522 | -0.013 (-0.044,0.017) | 0.584 | 0.002 (-0.037,0.040) | 0.929 |
| **RYR3** | T1 | 0 |  | 0 |  | 0 |  |
|  | T2 | -0.009 (-0.040,0.022) | 0.678 | -0.021 (-0.051,0.009) | 0.427 | -0.030 (-0.068,0.008) | 0.468 |
|  | T3 | -0.006 (-0.037,0.025) | 0.759 | -0.012 (-0.042,0.018) | 0.58 | -0.023 (-0.061,0.015) | 0.552 |

β coefficients represent the adjusted difference in BMD (g/cm²) relative to the lowest tertile (reference category). Model adjusted by age, BMI, ALT, AST, HDL, uric acid, serum vitamin D concentration, total energy intake, calcium intake, vitamin D intake, calcium supplementation and total cholesterol. *P* values were adjusted using the Benjamini–Hochberg false discovery rate (FDR) procedure.

**Supplementary Table 2.** Combined models of selected proteins to improve the classification of individuals with LBMD and NBMD.

| **Statistical Metrics** | **APOA1**  **+**  **SHGB** | **RYR1**  **+**  **APOA1** | **GSN**  **+**  **APOA2** | **GSN**  **+**  **SHGB** | **RYR1**  **+**  **SHGB** |
| --- | --- | --- | --- | --- | --- |
| **LBMD** | 80 | 82 | 215 | 216 | 217 |
| **NBMD** | 147 | 153 | 199 | 196 | 198 |
| OOB estimate of error rate (%) | 18,35 | 21,34 | 20,42 | 19,79 | 19,31 |
| Accuracy | **0,8406** | **0,831** | **0,816** | 0,7742 | **0,816** |
| 95% CI | (0.7326, 0.9176) | (0.7234, 0.9095) | (0.7368, 0.8796) | (0.6904, 0.8444) | (0.7368, 0.8796) |
| No Information Rate | 0,7246 | 0,7324 | 0,52 | 0,5242 | 0,504 |
| P-Value [Acc > NIR] | **0,01774** | **0,03626** | **4,99E-12** | **7,92E-09** | **4,30E-13** |
| Kappa | 0,58 | 0,6081 | **0,6331** | 0,5473 | **0,6322** |
| Mcnemar's Test P-Value | 0,54649 | 0,14891 | 0,2109 | 1 | 0,4042 |
| Sensitivity | **0,8679** | **0,8269** | 0,7692 | 0,7627 | 0,7778 |
| Specificity | 0,75 | **0,8421** | **0,8667** | 0,7846 | **0,8548** |
| Pos Pred Value | **0,92** | **0,9348** | 0,8621 | 0,7627 | 0,8448 |
| Neg Pred Value | 0,6316 | 0,64 | 0,7761 | 0,7846 | 0,791 |
| Precision | **0,92** | **0,9348** | 0,8621 | 0,7627 | 0,8448 |
| F1 | **0,8932** | 0,8776 | 0,813 | 0,7627 | 0,8099 |

Bold values indicate the greatest value for each metric. CI: confidence interval. Balanced accuracy = (sensitivity + specificity)/2. Protein concentrations were adjusted by age, BMI, ALT, AST, HDL, uric acid, serum vitamin D concentration, total energy intake, calcium intake, vitamin D intake, and calcium supplementation.  *Training data was obtained using matrix confusion.  Normal-Bone Mineral Density (NBMD), Low-Bone Mineral Density (LBMD).

**Supplementary Table 3.** Combined models of selected proteins to improve the classification between individuals with NBMD and osteopenia cases.

| **Statistical Metrics** | **APOA1**  **+**  **SHGB** |
| --- | --- |
| **OS** | 68 |
| **NBMD** | 147 |
| OOB estimate of error rate (%) | 23.33 |
| Accuracy | **0.9077** |
| 95% CI | (0.8098, 0.9654) |
| No Information Rate | 0.7846 |
| P-Value [Acc > NIR] | **0.007553** |
| Kappa | 0.7525 |
| Mcnemar's Test P-Value | 0.220671 |
| Sensitivity | **0.902** |
| Specificity | **0.9286** |
| Pos Pred Value | 0.9787 |
| Neg Pred Value | 0.7222 |
| Precision | 0.9787 |
| F1 | 0.902 |

Bold values indicate the greatest value for each metric. CI: confidence interval. Balanced accuracy = (sensitivity + specificity)/2. Protein concentrations were adjusted by age, BMI, ALT, AST, HDL, uric acid, serum vitamin D concentration, total energy intake, calcium intake, vitamin D intake, and calcium supplementation.  *Training data was obtained using matrix confusion.  Normal-Bone Mineral Density (NBMD), Osteopenia (OS).

**Supplementary Figure 1**. Receivers operate curves for the selected candidate biomarkers among LBMD vs. NBMD. The AUC values and optimal cut-off points are based on raw protein abundance data.
